# Pancreaticoduodenectomy for locally advanced colon cancer in hereditary nonpolyposis colorectal cancer

**DOI:** 10.1186/s12957-015-0755-7

**Published:** 2016-01-15

**Authors:** Rebecca Zhu, Gabriella Grisotti, Ronald R. Salem, Sajid A. Khan

**Affiliations:** 1 Yale University School of Medicine, New Haven, CT USA; 2 Department of Surgery, Yale University School of Medicine, New Haven, CT USA; 3 Department of Surgery, Section of Surgical Oncology, Yale University School of Medicine, PO BOX 208062, New Haven, CT USA

**Keywords:** Colorectal cancer, Early onset colorectal cancer, Locally advanced colorectal cancer, Hereditary nonpolyposis colorectal cancer, Lynch syndrome, Pancreaticoduodenectomy, Whipple procedure

## Abstract

**Background:**

Hereditary nonpolyposis colorectal cancer (HNPCC), or Lynch syndrome, accounts for 3 % of newly diagnosed cases of colorectal cancer. While a partial or subtotal colectomy is indicated for early stage disease, there is a paucity of data addressing locally advanced disease involving the foregut.

**Case presentation:**

We report two patients with hereditary nonpolyposis colorectal cancer presenting with locally advanced colon cancer surgically managed by pancreaticoduodenectomy with en bloc partial colectomy and a review of the literature.

**Conclusions:**

Locally advanced colorectal cancer in HNPCC is a rare clinical entity that requires special surgical consideration. Multidisciplinary treatment, including multi-visceral resection, offers the best long-term outcome.

## Background

Colorectal cancer (CRC) associated with hereditary nonpolyposis colorectal cancer (HNPCC) accounts for 3 % of newly diagnosed cases. It is genetically driven by an autosomal dominant mutation of DNA mismatch repair (MMR) genes and has a phenotypic penetrance of 70–80 % [1]. While a partial or subtotal colectomy may be indicated in early stage cancers, there is a paucity of data addressing locally advanced disease involving the foregut. We report two cases and review the literature in the management of this rare clinical entity.

## Case presentation

### Case no. 1

A healthy 56-year-old male presented with a 6-month history of early satiety, fatigue, and 80-pound weight loss. His mother and brother died from CRC at ages 36 and 44, respectively, and sister died from endometrial cancer. Physical exam revealed a firm, right upper quadrant mass.

Laboratory data included a hematocrit level of 32.7 % (normal 40–52 %) and carcinoembryonic antigen (CEA) level of 50.28 ng/dL (normal <3.0 ng/dL). Computed tomography (CT) of the chest, abdomen, and pelvis revealed a 13.0 × 10.0 cm localized right upper quadrant mass arising from the ascending colon proximal to the hepatic flexure, indistinguishable from the duodenum, and invading the right lateral abdominal wall. Colonoscopy revealed a large, nearly obstructing mass proximal to the hepatic flexure, and esophagogastroduodenoscopy showed a mass in the duodenum. Biopsies from both endoscopies revealed mucinous lesions suspicious but not diagnostic of adenocarcinoma. Neither the positron emission tomography (PET) nor the pre-operative laparoscopy showed evidence of metastatic disease. Neoadjuvant chemotherapy was not pursued due to lack of histologic confirmation of malignancy. Prior to surgery, he was admitted with a lower extremity deep vein thrombosis (DVT), for which he received an inferior vena cava (IVC) filter and therapeutic anticoagulation with enoxaparin.

The patient underwent a right hemicolectomy with en bloc classic pancreaticoduodenectomy and resection and reconstruction of the anterior abdominal wall. Reconstruction involved an end-to-side pancreaticojejunostomy, hepaticojejunostomy, gastrojejunostomy, and ileocolostomy. He had an unremarkable post-operative course. Histopathology of the surgical specimen revealed moderately differentiated mucinous adenocarcinoma arising from the hepatic flexure of the colon with focal invasion into a segment of the adjacent small bowel, peripancreatic soft tissues, and duodenum. Seventy-six lymph nodes and all surgical resection margins were negative for tumor. Immunohistochemistry of the surgical specimen showed absence of DNA mismatch repair enzymes hMLH1 and hPMS2 with retention of normal hMSH2 and hMSH6. Polymerase chain reaction (PCR) was consistent with high microsatellite instability (MSI-H) in the tumor. Genetic testing of peripheral blood leukocytes was consistent with mutated hMLH1. He underwent adjuvant chemotherapy with 12 cycles of oxaliplatin, fluorouracil, and folinic acid (FOLFOX) chemotherapy. He remains free of disease 4.5 years after surgery.

### Case no. 2

**Fig. 1:**
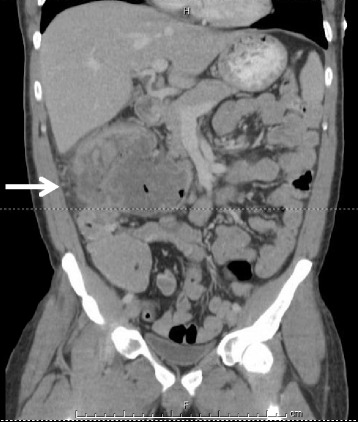
Computed tomography with intravenous and oral contrast from case 2. Coronal section of the abdomen and pelvis. *Arrow* points to 14.0 × 9.8 × 10.8 cm heterogeneous mass in the right upper quadrant abutting the ascending colon and duodenum. Oral contrast is seen within the mass. The fat plane between the mass and the second segment of the duodenum is obscured

**Fig. 2:**
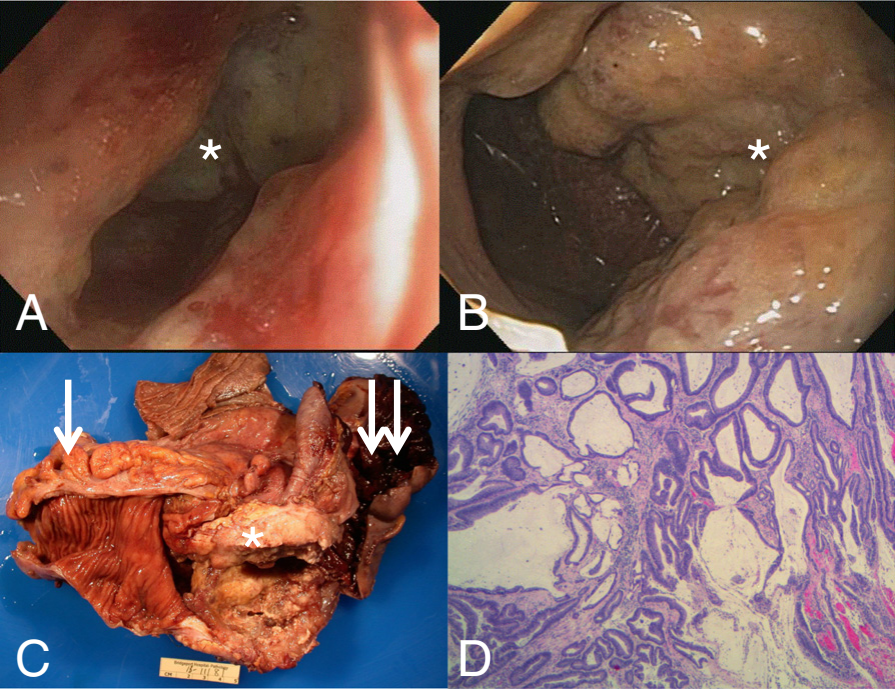
Endoscopy images, gross pathology, and histopathology from case 2. **a** Colonoscopy revealed an ulcerated, partially obstructing, large mass in the transverse colon (marked by *asterisk*). The pediatric colonoscope could not completely traverse this large mass due to tumor obstruction and alteration in the normal colon anatomy. **b** Upper endoscopy showed a large malignant ulcerated mass (marked by *asterisk*) with no bleeding in the second part of the duodenum. **c** Part of the surgical specimen including colon (marked by *single arrow*), duodenum (marked by *double arrow*), with the mass (marked by *asterisk*) with a diffusely mucinous appearance. **d** Hematoxylin and eosin stain of primary colonic adenocarcinoma (viewed at ×40) with large pools of mucin evident. The tumor was 14 cm, low grade mucinous adenocarcinoma of the colon with loss of mismatch repair proteins and high microsatellite instability. There was mild to moderate intratumoral lymphocytes, mild to moderate peritumor lymphocytes, with low grade tumor budding, and no lymphovascular invasion. Margins were negative and 22 lymph nodes were negative (not shown)

A healthy 36-year-old male presented with fatigue, vague abdominal pain, diarrhea, and unintentional 40-pound weight loss. He was profoundly anemic with hemoglobin 4.2 g/dL (normal 13.0–18.0 g/dL), and CT demonstrated a 14-cm heterogeneous mass in the right hemi-abdomen (Fig. 1). Colonoscopy revealed a partially obstructing tumor near the hepatic flexure (Fig. 2a), biopsies were consistent with adenocarcinoma, and immunohistochemistry revealed the absence of DNA mismatch repair enzymes hMSH2 and hMSH6 with retention of normal hMLH1 and hPMS2. Upper endoscopy revealed a 7-cm partially obstructing ulcerated mass in the second part of the duodenum (Fig. 2b); biopsies revealed a well-differentiated adenocarcinoma with immunostains suggesting a colonic primary (CK20/CDX2-positive, CK7-negative). Tumor markers were remarkable for an elevated CEA level of 46.3 ng/dL (normal <3.0 ng/dL). Neoadjuvant chemotherapy with oxaliplatin, fluorouracil, iniontecan, and leucovorin (FOLFIRINOX) was administered but the patient only received 3 cycles. This treatment was complicated by gastrointestinal bleeding and bilateral pulmonary emboli after bilateral lower extremity DVTs. Anticoagulation was initially contraindicated, so an IVC filter was placed. The third cycle of chemotherapy was complicated by a perirectal abscess requiring drainage. Poor nutritional parameters prompted aggressive enteral feeds via a nasojejunal feeding tube (pre-albumin 16 mg/dL (normal 18.5–35.8 mg/dL), albumin 2.8 g/dL (normal 3.4-5.4 g/dL)). His CEA level decreased to 22.7 ng/dL (normal <3.0 ng/dL).

The patient underwent a pylorus sparing pancreaticoduodenectomy with en bloc right hemicolectomy. Reconstruction involved an end-to-side pancreaticojejunostomy, end-to-side hepaticojejunostomy, end-to-side duodenojejunostomy, and anatomical side-to-side and functional end-to-end ileocolostomy. Examination of the specimen revealed mucinous adenocarcinoma arising from the right colon with direct extension to the duodenal mucosa (Fig. 2c, d), and histopathology showed absence of DNA mismatch repair and high microsatellite instability (MSI-H). Twenty-two lymph nodes and all surgical resection margins were negative for tumor. There was no definitive tumor response to treatment. Post-operative course was remarkable only for a grade A pancreatic fistula that was successfully managed by percutaneous drainage and subcutaneous octreotide. He was discharged on post-operative day 8 on rivaroxaban in light of his continued need for anticoagulation. The patient completed 4 months of adjuvant chemotherapy and remains well without evidence of recurrence 2 years after surgery.

### Discussion

Colorectal cancer (CRC) is the third most common malignancy and third leading cause of cancer death in the USA, with around 136,830 estimated new cases in 2013. There are several well-defined inherited CRC syndromes that include HNPCC, familial adenomatous polyposis (FAP), *MutY* human homologue-associated polyposis (MAP), Peutz-Jeghers syndrome, and Juvenile polyposis syndrome [2]. These syndromes are the result of highly penetrant germ line mutations that predispose to an early age onset and high lifetime risk of developing CRC.

HNPCC is the most common of all identified hereditary CRC syndromes with a mean age of colon cancer diagnosis of 61 years. Loss of expression of mismatch repair genes (*MLH1*, *MSH2*, *MSH6*, *PMS2*) secondary to mutations is a characteristic of HNPCC and accounts for over 97 % of mutations [3]. Recently, germ line mutations in the *EPCAM* gene that inactivate *MSH2* have also been identified in families of HNPCC patients [3, 4]. These genetic abnormalities are characterized by amplifications of microsatellite loci throughout the genome resulting in microsatellite high instability (MSI-H). MSI-H is histologically characterized by tumor-infiltrating lymphocytes, Crohn’s like lymphocytic reaction, and mucin; tumors are unique in that they are predominantly proximal to the splenic flexure, have a low likelihood for regional and distant metastasis, and the adenoma-to-carcinoma sequence is accelerated to 2–3 years compared to 7–10 year progression seen in sporadic cases [4]. Furthermore, HNPCC is also associated with a predisposition to the development of extra-colonic malignancies arising from intra-peritoneal organs, as well as the brain and skin. Therefore, appropriate management of locally advanced CRC in the context of HNPCC requires special consideration.

For diagnosis, according to the National Comprehensive Cancer Network (NCCN) guidelines, all patients diagnosed with colorectal cancer under the age of 70, or over the age of 70 and who also meet the Bethesda Guidelines, should undergo tumor testing by immunohistochemistry or MSI testing. Greater than 90 % of HNPCC tumors are MSI-H and/or lack expression of at least one mismatch repair protein, so while positive screening tests increase the possibility of HNPCC in younger patients, a definitive diagnosis cannot be made without identifying a germ line mutation [2]. Workup of newly suspected HNPCC cases should be similar to all newly diagnosed cases of colon cancer. This includes a complete colonoscopy to rule out synchronous lesions, staging CTs of the chest, abdomen, and pelvis, and serum CEA to guide post-resection surveillance. Though not recommended for sporadic colon cancer, strong consideration for an upper endoscopy and transvaginal ultrasound should be made because of heightened risks for the development of upper gastrointestinal malignancies and gynecologic malignancies, respectively.

With regard to surgical management, a subtotal colectomy or segmental resection should be offered to patients. Given the measurable compromises to long-term bowel function and quality of life, without clear survival advantage, our group favors a segmental colectomy and regional lymphadenectomy as opposed to subtotal colectomy [5, 6]. In the cases presented in this paper, genetic testing and confirmation of HNPCC was performed post-operatively, so while there was a high suspicion for HNPCC during pre-operative evaluation, surgical decision-making was performed in the context of locally advanced CRC with potentially curative resection. For these cases, much has been written about locally advanced, sporadic CRC with invasion into the pancreatic head or duodenum requiring pancreaticoduodenectomy. Multi-visceral resection achieves an oncologically sound operation associated with prolonged overall survival, reduced local recurrence rates, and acceptable perioperative morbidity and mortality when performed by experienced surgeons [7, 8]. Recently, investigators have reported that a more extensive en bloc pancreaticoduodenectomy compared to an en bloc local resection is associated with an improved 5-year overall survival [9]. However, few reports address this surgical scenario in the context of HNPCC. While the basic oncologic principles of primary resection still apply, 25 % of HNPCC patients who undergo a segmental colectomy will develop metachronous CRC after the index diagnosis [10]. This is why some surgeons advocate for an upfront total abdominal colectomy and ileorectal anastomosis, but there has yet to be any data demonstrating an improvement in survival in patients undergoing such an approach [6]. A prophylactic total or subtotal colectomy in the context of a multi-visceral resection would seem unnecessary. Regardless, in non-emergent cases, the benefits and risks of local vs extended resection should be discussed with patients who are MMR deficient with confirmatory genetic testing, keeping in consideration patient preference, the effect of bowel function on quality of life, and the predicted post-operative compliance to surveillance.

The current standard for adequate staging of right-sided colon cancers remains at least 12 lymph nodes, as espoused by the NCCN [2]. Interestingly, MSI-H is associated with higher lymph node yields in a prospective study of 204 patients with colon cancer, stages I–III (67 % of patients had ≥12 lymph nodes sampled, while 79 % of patients with microsatellite instability had ≥12 lymph nodes sampled (OR 2.5, *p* = 0.007)); 5.6 % of the total lymph nodes were positive for metastasis [11] compared with 46 % of microsatellite stable (MSS) CRC resections in a separate study [12]. This raises the question of whether the proportion of positive lymph nodes may be a better prognosticator than the absolute number [13]. However, the significant values for such lymph node ratios are still under investigation and even with a ratio, there will still need to be an absolute minimum of lymph nodes recommended for retrieval.

The paradigm of chemotherapy for locally advanced CRC is changing. Recommendations for the use of pre-operative neoadjuvant chemotherapy to provide better local control for R0 resection have been increasing, citing the theoretical eradication of micrometastases and reduced perioperative tumor shedding, as well as downstaging with better progression-free and overall survival. More recently, there is evidence of added benefit in combining neoadjuvant and adjuvant treatment for CRC, as was performed in case 2 [14]. However, the benefit of chemotherapy in patients with HNPCC, regardless of timing, is unclear. Because of the distinct molecular background of MSI-H tumors, it has been suggested that they may be less responsive to traditional CRC chemotherapeutic agents. In a retrospective study from the Netherlands, there was no difference in 5-year overall survival in patients with stage III colon cancer of HNPCC families in comparing patients treated with and without adjuvant 5FU (70 % 5-year survival for both the group that received adjuvant therapy and the group that did not) [15]. The benefit of chemotherapy in MSI-H CRC in HNPCC requires further investigation.

## Conclusions

Our two cases have several striking similarities that highlight important issues in the surgical management of HNPCC. Each patient presented with proximal, locally advanced, non-metastatic CRC. Careful pre-operative planning was required to perform multi-organ, en bloc resections with negative surgical margins. Despite aggressive lymphadenectomies, neither patient had lymph node metastases arising from their MSI-H tumor. After completing adjuvant chemotherapy, each patient remains disease free at 4.5 and 2.0 years from surgery, respectively. Intriguingly, both patients were also diagnosed with DVTs prior to surgery. However, there is little information available on whether HNPCC confers a risk of hypercoagulability.

Locally advanced CRC involving the foregut is a rare presentation of HNPCC. A well-planned, multi-modality treatment approach is necessary for treatment. A review of the literature, summarized in Table 1, supports that a pancreaticoduodenectomy with en bloc segmental colectomy and regional lymphadenectomy provides patients with the potential for cure.

**Table 1:** Comparison of studies with regards to interventions for colorectal cancer

Authors	Year	Number of patients	Intervention evaluated	Main conclusions
You et al.	2008	522	Quality of life (QOL) after extended (201) vs segmental (321) colon resection	Extended resection yielded compromised bowel function, with decreased QOL
Zhang et al.	2013	14	En bloc pancreaticoduodenectomy (PD) and right hemicolectomy in right colon cancer	Outcomes no worse than stage-matched patients without adjacent organ involvement
Temple et al.	2014	635	PD (607) vs PD with colon resection (28)	No significant difference in post-operative mortality
Cirocchi et al.	2014	53	En bloc (39) vs local resection (14) in locally advanced right colon cancer	En bloc resection improved overall 5-year survival
Kalady et al.	2010	296	Segmental (253) vs total colectomy (43) in HNPCC	Segmental resection increases the risk of metachronous colon cancer
Berg et al.	2013	204	Impact of tumor genetics on lymph node harvest in stage I–III colon cancer	Node harvest was greatest for cancers with MSI but without KRAS/BRAF
Samdani et al.	2015	256	Effect of mismatch repair deficiency on lymph node yield in colorectal cancer	Mismatch repair deficiency did not predict lymph node yield
Sugimoto et al.	2015	4172	Prognostic value of lymph node ratio in stage III colon cancer	Lymph node ratio, with a cutoff value of 0.18, was a significant independent prognostic factor
Hong et al.	2014	321	Comparison of adjuvant chemotherapy (fluorouracil and leucovorin (161) vs FOLFOX (160)) in stage II–III rectal cancer after neoadjuvant chemoradiotherapy and resection	Adjuvant FOLFOX improved survival compared to adjuvant fluorouracil and leucovorin
de Vos tot Nederveen Cappel et al.	2004	92	Effect of adjuvant therapy with 5-FU (28) vs no adjuvant therapy (64) in stage III colon cancer of HNPCC	5-year survival did not differ

### Consent for publication

We have consent to publish from the individual patients whose cases were presented in this report.

## Abbreviations

CEA: carcinoembryonic antigen
CRC: colorectal cancer
CT: computed tomography
DVT: deep vein thrombosis
FAP: familial adenomatous polyposis
FOLFOX: oxaliplatin, fluorouracil, and folinic acid
HNPCC: hereditary nonpolyposis colorectal cancer
IVC: inferior vena cava
MAP: *MutY* human homologue-associated polyposis
MMR: mismatch repair
MSI: microsatellite instability
MSI-H: high microsatellite instability
MSS: microsatellite stability
NCCN: National Comprehensive Cancer Network
OR: Odds ratio
PCR: polymerase chain reaction
PD: pancreaticoduodenectomy
PET: positron emission tomography
QOL: quality of life
